# Canadian Association of Gastroenterology: Strategic Plan 2016–2020

**DOI:** 10.1155/2016/5301452

**Published:** 2016-07-19

**Authors:** Derek McKay, David Armstrong, Daniel Sadowski, Nicola Jones, Wallace MacNaughton, Paul Sinclair

**Affiliations:** ^1^University of Calgary, Calgary, AB, Canada T2N 4N1; ^2^McMaster University, Hamilton, ON, Canada L8S 4K1; ^3^University of Alberta, Edmonton, AB, Canada T5H 3V9; ^4^University of Toronto, Toronto, ON, Canada M5G 1X8; ^5^Canadian Association of Gastroenterology, Oakville, ON, Canada L6J 7W5

The CAG is a professional organization that seeks to promote the discipline of gastroenterology in Canada and internationally through a series of activities aligned with a Strategic Plan (renewed and updated every five years) in order to advance our mandate of (i) supporting and engaging in the study of the organs of the digestive tract in health and disease, (ii) promoting the advancement of the science and art of gastroenterology by providing leadership in patient care, research, education, and continuing professional development (CPD), and (iii) promoting and maintaining the highest ethical standards (https://www.cag-acg.org/about/what-is-the-cag/).

Structurally, the Board of Directors (President, President-Elect, Past President, VP Treasurer, and VP Secretary) provides oversight governance of the CAG, while operational functions are performed by the Executive Director, the CAG National Office Staff, and five Vice-Presidents (Administrative Affairs, Clinical Affairs, Education Affairs, Quality Affairs, and Research Affairs (Operations Committee (OC))). The OC recruits CAG members to serve on standing committees or dedicated taskforces directed towards implementation of CAG's Strategic Plan. The Canadian Digestive Health Foundation (CDHF) is the CAG's Foundation with the CAG Board members being the only voting members of the CDHF [1]. The CAG Board provides oversight and general direction to the CDHF Governing Board of Directors to ensure alignment with the CAG's Strategic Plan.

With a new governance structure in place, the 2010–2015 Strategic Plan was implemented with remarkable success as presented in the CAG news page [2]. For instance, surveys of perceived learning needs of the membership identified the need for improved quality in endoscopic skills and endoscopic teaching of trainees. This led to the development of the highly successful Skills Enhancement in Endoscopy© (SEE©) programme that will be a flagship programme for years to come. Similarly, education is a major component of the CAG mandate and CAG as an accrediting body is a recognized leader in accreditation amongst national associations/societies in Canada. Canadian Digestive Diseases Week*™* is the flagship educational event for CAG, in collaboration with the CASL, with increasing numbers of delegates each year.

The CAG Board and OC exist solely to serve the needs of the members of the CAG; the CAG provides regular updates on the CAG and the CDHF activities by monthly emails to all members (e-blasts), a frequently updated website (https://www.cag-acg.org/), an annual report, and reporting at the annual general meeting at CDDW. Communication with the membership is central to the success of the CAG. At the outset of the 2010–2015 CAG Strategic Plan a cross-cutting communication strategy was introduced to ensure that the membership is informed about all the CAG activities and that the leadership of the CAG receives input from all constituencies within the membership: clinicians, clinician scientists, discovery researchers, trainees, junior staff, and established academics or practitioners.

Over the last fifteen months, the CAG Board has solicited opinions from the membership (two surveys, individual discussions), the Past-Presidents Council, and the OC regarding (1) the CAG's areas of strengths and weakness, (2) concerns relating to career opportunities and development, clinical practice, and research, and (3) the future of gastroenterology in Canada. Integrating this information and building on the success of 2010–2015 Strategic Plan, the CAG Board is pleased to present a roadmap for the continued success of the organization that will be operationalized in the 2016–2020 CAG Strategic Plan.

Before outlining the plan, the CAG Board takes this opportunity to communicate directly with the CAG membership: the CAG is your association and as a volunteer organization, its success and productivity depend on you. There are many ways to get involved and support the association; we encourage you to be proactive and contact the National Office if you have an area of interest and to contribute if you are asked to assist with an initiative. Finally, the Strategic Plan is an evolving document and we would appreciate your input now or at any time over the next five years.

*Goal  1*. The CAG and the CDHF will continue to advocate for and facilitate the development of a renewed, larger, and forward-looking vision of digestive healthcare and will, where appropriate, partner with allied digestive healthcare stakeholders to deliver on this goal. This will be accomplished by

- enhancing public awareness of digestive health and developing communications strategy, involving CAG's foundation, the CDHF, that will keep digestive health issues in the public eye and on the agenda of stakeholders;
- working with national and provincial associations to identify the Canadian burden of GI illness, challenges facing the gastroenterologist, and workforce issues that are critical to optimal delivery of patient care;
- being recognized as a leader in the development of digestive healthcare policy at a national level.

*Goal  2*. The CAG will focus on attracting new members while engaging, retaining, and adding value for the existing membership of gastroenterologists, digestive health researchers, and the digestive health constituency by

- developing and maintaining a data base of digestive health service providers;
- ascertaining and addressing the needs of the digestive health services constituency;
- anticipating the need for and leading in the implementation of modifications to training and CME to ensure the members are prepared for the changing landscape of digestive health;
- identifying and implementing strategies to support development of clinician scientists;
- facilitating acquisition of leadership skills and encouraging diversity in the CAG leadership and membership.

*Goal  3*. The CAG will be the preeminent accredited provider of continuing education for our members and other digestive healthcare providers to improve the quality of digestive healthcare for all Canadians by

- incorporating the principles of competency-based medical education into the identification of perceived and unperceived gaps in learners' knowledge and skills;
- working with key partners and stakeholders to define competencies and standards required for the delivery of high quality digestive healthcare;
- developing innovative strategies, including point-of-care monitoring and network-based evaluation of healthcare delivery, to measure the quality of digestive healthcare across Canada;
- keeping pace with the evolving discipline and practice of gastroenterology to ensure that CAG members are provided with world-class educational events and opportunities for skills enhancement in research and clinical practice;
- continuing to develop and publish authoritative, evidence-based guidelines to support the delivery of high quality digestive healthcare;
- becoming a key provider of knowledge transfer through the effective and appropriate delivery and evaluation of educational programs;
- facilitating the maintenance of certification program through a simple, integrated, and streamlined system.

*Goal  4*. The CAG will place a high priority on supporting and facilitating discovery and clinical research during training and directly supporting individual investigators, teams, and networks. This will be accomplished by

- targeting efforts from the CAG and the CDHF to support existing strengths in Canada in discovery biomedical research and to develop and expand expertise in patient-oriented/directed research and health economics and policy by supporting trainees (students, postdoctoral fellows, and residents) and by providing operating grants and career support awards;
- developing an infrastructure to facilitate team- and network-based research to capitalize on perceived future funding opportunities and development of a pan-Canadian network that can integrate and optimize the combined and synergistic efforts of clinical and basic scientists;
- supporting and promoting clinical researchers, providing directed and targeted support to enhance their chances of success, and working to help overcome the barriers that can forestall the critical contribution that such individuals make to the advances in the discipline in gastroenterology;
- aligning with partners to ensure that research outcomes are transmitted to the appropriate stakeholders and that knowledge is transferred in a timely fashion to elicit increased awareness of the burden of digestive disease and the mechanisms of digestive disease and to advance the pace at which discoveries can be implemented to enhance patient care.

*Goal  5*. The CAG will anticipate and plan for future developments in digestive healthcare. Specific steps will be taken to ensure that our members have access to appropriate training, new therapies, and technology to better care for our patients. This will be accomplished through

- monitoring of emerging areas such as nutrition, the microbiome, “big data,” novel therapeutics and diagnostic technologies;
- fostering collaborative research and member/trainee education in emerging areas;
- assessing the effectiveness of new technologies and providing expert guidance regarding utilization;
- encouraging cooperation between payers and industry to ensure optimal patient access to new therapeutics;
- utilizing the power of emerging information technology to accelerate the transition from bench to bedside.

The CAG has had many remarkable successes to date. Implementation of the new five-goal plan will keep the CAG at the forefront of gastroenterology in Canada with an increasing presence on the international stage. There are significant challenges ahead including shrinking funds for research; changing directives from the RCPSC and physician competency regulations; changing interactions with pharmaceutical and not-for-profit agencies; and the introduction of new technologies for training and for enhancing patient care. The CAG 2016–2020 Strategic Plan recognizes these challenges and works to the CAG's established strengths within a vision that the CAG will remain the leading provider of accredited educational activities in gastroenterology in Canada and is looking to the future to ensure that the membership is aware of innovations in the field that will impact training and the discipline of gastroenterology (e.g., new diagnostics, diet and nutrition, and microbiome in health and disease). Within this Strategic Plan, the activities of the CDHF, interactions with our established (and new) partners, and a constant vigilance on quality initiatives will be cornerstone activities.

The CAG 2016–2020 Strategic Plan is determined by the CAG Mission Statement, builds on the strengths and achievements of past strategic plans, and is visionary with a view for the future (e.g., development of research networks) but also sets tangible and achievable goals. Achieving these goals demands commitment and dedication from the CAG leadership and membership. The CAG Board of Directors welcome all comments from the CAG membership on the activities of the CAG and the implementation of this Strategic Plan: a plan that can be both flexible and responsive to meet the needs of the members and changes within the Canadian landscape as they relate to gastroenterological research, the practice of gastroenterology, the care of patients, and the promotion of digestive health.

*Derek McKay* *David Armstrong* *Daniel Sadowski* *Nicola Jones* *Wallace MacNaughton* *Paul Sinclair*
